# Sensitivity to Instruction Strategies in Motor Learning Is Predicted by Anterior–Posterior TMS Motor Thresholds

**DOI:** 10.3390/brainsci15060645

**Published:** 2025-06-16

**Authors:** Michael L. Perrier, Kylee R. Graham, Jessica E. Vander Vaart, W. Richard Staines, Sean K. Meehan

**Affiliations:** Department of Kinesiology and Health Sciences, University of Waterloo, 200 University Ave W, Waterloo, ON N2L 3G1, Canada; mperrier@uwaterloo.ca (M.L.P.); krgraham@uwaterloo.ca (K.R.G.); jevandervaart@uwaterloo.ca (J.E.V.V.); rstaines@uwaterloo.ca (W.R.S.)

**Keywords:** transcranial magnetic stimulation (TMS), explicit, implicit, plasticity, motor control, skill learning

## Abstract

**Background:** The impact of exogenous explicit knowledge on early motor learning is highly variable and may be influenced by excitability within the procedural sensorimotor network. Recent transcranial magnetic stimulation (TMS) studies suggest that variability in interneuron recruitment by anterior–posterior (AP) currents is linked to differences in functional connectivity between premotor and motor regions. **Objectives:** This study used controllable pulse parameter TMS (cTMS) to assess how AP-sensitive interneuron excitability interacts with explicit knowledge to influence motor learning. **Methods**: Seventy-two participants were grouped as AP-positive (n = 36) and AP-negative groups (n = 36) based on whether an AP threshold could be obtained before reaching maximal stimulator output. A narrow (30 µs) stimulus was employed to target the longest latency corticospinal inputs selectively. Participants then practiced a continuous visuomotor tracking task and completed a delayed retention test. Half of each group received explicit knowledge of a repeated sequence embedded between random sequences. Random sequence tracking performance assessed general sensorimotor efficiency; repeated sequence performance assessed sequence-specific learning. **Results**: Both AP_30_-positive participants, with and without explicit knowledge, and the AP_30_-negative without explicit knowledge demonstrated similar improvements in sensorimotor efficiency driven by offline consolidation. However, AP_30_-negative participants given explicit instruction exhibited significantly reduced improvement in sensorimotor efficiency, primarily due to impaired offline consolidation. **Conclusions**: These findings suggest that individuals with low excitability in long-latency AP-sensitive inputs may be more vulnerable to interference from explicit instruction. The current results highlight the importance of accounting for individual differences in interneuron excitability when developing instructional strategies for motor learning.

## 1. Introduction

The processes governing learning and memory are subdivided into two broad systems, termed declarative and procedural [1,2,3]. The declarative system, spanning the medial temporal lobe and areas of the neocortex [4,5], supports the acquisition of explicit, factual knowledge about motor skills. In contrast, the procedural system, spanning the basal ganglia, cerebellum, premotor areas, and motor cortex [4,5], supports the acquisition of implicit skilled knowledge through physical practice without conscious awareness [6]. Historically, the two memory systems were thought to operate independently [7]. However, contemporary evidence suggests that the two memory systems operate in a more complex parallel manner, where the two systems interact and may compete for neural resources during skilled performance and learning [8,9].

A common recurring theme is how explicit processes contribute to all forms of motor learning, particularly during the early stages of learning [10]. During the initial stages of skill acquisition, explicit instruction can be derived from an external source (i.e., coach, instructor, or therapist) or autogenously acquired through the learner’s own experience. However, behavioral evidence is conflicted regarding the benefits of explicit knowledge to motor learning within and across healthy [11,12,13] and older populations [14,15,16]. The main question is what neural mechanism(s) drive this variability.

The neural mechanisms governing motor control and learning are mediated by several sensorimotor loops that converge on corticospinal neurons in the primary motor cortex to shape efferent output to the muscles [17]. During the early stages of skill acquisition, a broad set of loops spanning cerebral and subcortical structures, including the striatum, cerebellum, motor cortical regions, and parietal cortices, in addition to frontal associative areas and limbic areas [18]. As skilled ability increases, the rate of learning becomes incremental, and the relative contributions of different sensorimotor loops become more focused and distinct [18]. Motor skill learning more heavily recruits the cortico-striatal loop, while motor adaptation more heavily recruits the cortico-cerebellar loop [18]. In addition, frontal associative regions and limbic areas are recruited to a lesser extent as learning shifts from explicit testing of strategic hypotheses to developing sensorimotor efficiency and automaticity [19].

A safe, non-invasive approach to probe the functional contribution of the various sensorimotor loops to motor control and learning is to quantify one or more phenomena elicited using transcranial magnetic stimulation (TMS) [20]. TMS uses time-varying magnetic fields to produce electric currents in the brain [21]. When a single TMS stimulus is applied over the primary motor cortex, the induced current activates a series of excitatory transsynaptic inputs called indirect (I-) waves that converge on the corticospinal output neurons [22]. The specific set of transsynaptic inputs recruited by the TMS stimulus depends on stimulus characteristics such as the direction and duration of the current [23]. For example, the latency of the I-waves recruited by anterior–posterior (AP) induced currents is longer and more variable than the I-waves generated by posterior-anterior (PA) currents [24,25]. PA and AP-induced currents also demonstrate different time constants, indicating that they act on different cortical axons [23,25]. The onset latencies of the motor evoked potential (MEP) elicited by AP current are also longer and more variable for AP than for PA current [26]. TMS-EEG studies further support the recruitment of distinct sets of interneurons by PA and AP-induced current. PA and AP induced current over various cortical targets differentially elicit early transcranial evoked potential (TEP) components spanning 15–100 ms post-TMS that are localized in part to generators in the primary motor cortex [27,28].

The longer latency of the I-waves and MEP onset for AP current suggests the activation of a higher-order polysynaptic circuit [29]. In contrast, PA-induced current is thought to act on monosynaptic inputs to the corticospinal neuron [29]. The increased complexity of the circuits sensitive to AP current raises the possibility that these circuits underpin some of the variability across individuals in sensorimotor ability. For example, the variation in corticospinal inputs across individuals appears to be functionally significant [30]. While both PA and AP-induced currents may recruit inputs originating from premotor areas [30,31,32], only the variation in individual AP current response was associated with the functional connectivity of primary motor cortex with the ipsilateral premotor cortex and bilateral supplementary motor areas [30]. In all cases, individuals with the highest functional connectivity demonstrated the strongest propensity to recruit the AP-sensitive corticospinal inputs. Therefore, the excitability of the corticospinal inputs recruited by AP-induced current may indicate individual variations in motor control and learning. A more readily recruitable set of AP-sensitive inputs may help to balance or facilitate the transition between the declarative and procedural memory systems throughout the learning process.

The current study explored the effect of AP interneuron excitability and explicit instruction on performance and offline consolidation during the early learning phases. Specifically, we used controllable pulse parameter TMS (cTMS) [33] to selectively probe the excitability of the AP-sensitive circuits that take the longest time to influence corticospinal output. We hypothesized that participants with higher AP thresholds, indicative of reduced interneuron excitability, would exhibit reduced offline consolidation and overall performance gains when given explicit instruction compared to those with higher AP thresholds practicing under implicit conditions. In contrast, we hypothesized that practice’s explicit/implicit nature would be marginal for those with lower AP thresholds. We did not expect any differences between AP thresholds and the explicit/implicit nature of the task during practice.

There is increasing evidence that the AP current induced by traditional monophasic TMS stimulation (~70–82 µs) recruits a mix of functionally distinct inputs to the motor corticospinal neuron [23,34]. Narrowing the AP pulse duration to 30 µs (AP_30_) acts on interneuron axons with the longest-strength duration time constant and produces MEPs with longer latencies than wider pulse durations, whose time constants and MEP onset latencies are more consistent with PA pulses [23]. We hypothesized that these long-latency circuits recruited by AP_30_ current likely act as a modulatory influence on corticospinal neurons and that variation in their excitability would mediate motor learning. Further, we included random and repeated sequences within the continuous tracking task to assess whether any mediatory effect would influence general sensory motor ability and sequence-specific skill learning. Enhanced sensorimotor ability is reflected by improvements in random sequence tracking performance, while sequence-specific learning is quantified by greater improvements in repeated sequence tracking compared to random sequence tracking.

## 2. Materials and Methods

### 2.1. Participants

Seventy-two healthy adults (32 male, 40 female, 23.2 ± 4.1 years, mean ± standard deviation) with no history of neurological disease or contraindications to TMS were recruited. All participants provided written informed consent, and this study was approved by the University of Waterloo’s Clinical Research Ethics Board (ORE #45068).

### 2.2. Experimental Design and Procedure

**Figure 1:**
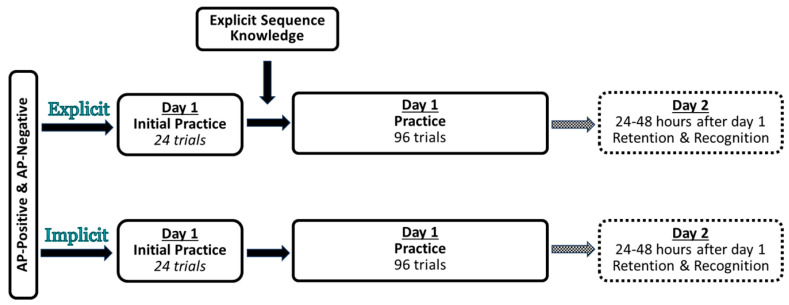
Experimental study design. Participants in the AP_30_-positive and AP_30_-negative groups were randomly assigned to an Implicit or Explicit group. Both groups completed two sessions that took place 24–48 h apart. The first session consisted of 24 trials of the continuous tracking task, followed by an additional 96 trials. The only difference between the implicit and explicit groups was that the explicit group received explicit knowledge about the repeated waveform after the initial practice in the first session.

The experiment consisted of a training session and a delayed retention test session 24 to 48 h later (Figure 1).

For the first session, participants completed a demographic questionnaire and were screened for contraindications to TMS. Participants were subsequently divided into two groups based on the ability to elicit a 0.5 mV MEP using AP_30_-induced current (Table 1). If a 0.5 mV MEP could be induced by the AP_30_ stimulus before reaching maximal stimulator output, the participant was assigned to the AP_30_-positive group. In contrast, if the participant’s 0.5 mV AP_30_ threshold exceeded maximal stimulator output, they were assigned to the AP_30_-negative group.

All participants performed two blocks of twelve continuous visuomotor tracking task trials following threshold determination. Each trial lasted 30 s. These two blocks served as the initial performance measure. Participants in the AP_30_-positive and AP_30_-negative groups were then randomly assigned to an implicit or explicit instruction group. All four groups then performed 96 trials of continuous visuomotor tracking split across eight blocks. The only difference between the implicit and explicit groups was that the AP_30_-positive and AP_30_-negative explicit groups were provided with explicit knowledge about a brief repeated sequence embedded within each trial of the continuous visuomotor task. A recognition test was performed before the eight blocks of practice to confirm that all participants in the explicit groups had gained explicit knowledge of the repeated sequence.

The delayed retention test session was identical for all groups and consisted of one block of 12 trials of the continuous tracking task followed by a recognition test.

### 2.3. Continuous Tracking Task

Participants were seated in a chair in front of a 24″ computer monitor (P2418HT, Dell Canada, North York, ON, Canada). Their arms were positioned on the table with a 90° bend at the elbow and the forearm pronated. The participant’s right index finger was positioned to rest against a thin film force-sensing resistor (RP-S40-ST, DFRobot, Shenzhen, China ) mounted to a block of wood anchored to the table.

**Figure 2:**
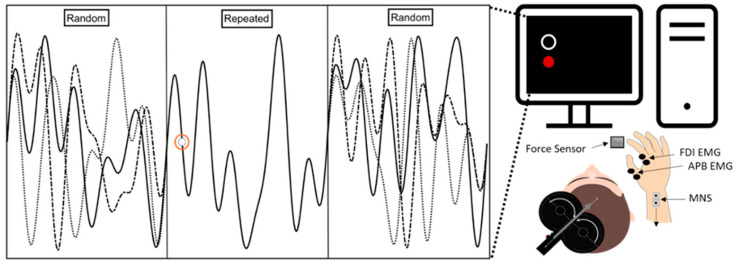
An example of the experimental setup and a description of the waveform movement across the screen. The participant was seated in front of a desk with a computer monitor, and their right arm rested on the table. The participant abducted their index finger against a force sensor to control the cursor’s vertical position (red dot), tracking the target (white circle). The solid and dashed lines illustrate the movement of the target across the random and repeated epochs for four trials. Applying greater force moved the cursor upwards, while decreasing the force moved it downwards. During the first session, SAI was assessed while participants performed the task. Electrodes were placed on the right first dorsal interosseous (FDI) and abductor pollicis brevis. Right median nerve stimulation (MNS) was delivered via a bar electrode with the cathode proximal. The participants only viewed the red dot and a white circle on a black background; they did not see any solid or dashed representations of the waveforms. The waveform consisted of random and repeated epochs. The random epochs changed across blocks, while the repeated epoch remained constant throughout the experiment.

The continuous tracking task was a variation of the task described by Wulf and Schmidt [35]. A red dot (target) and a white circle (cursor) moved from left to right on the computer screen at a fixed rate during the task. The red dot moved in a predetermined vertical sinusoidal pattern. The participant was instructed to track the red dot with the white circle by abducting their index finger against a force sensor (Figure 2). Increased force against the sensor caused upward cursor movement, while reduced force caused downward cursor movement. Before starting the initial practice, each participant’s force range was calibrated to span 0% to 20% of maximum voluntary contraction. A 10% of maximum voluntary contraction equated to the middle point of the vertical display. The cursors’ and targets’ positions were controlled using custom software (LabVIEW 2022, National Instruments, Austin, TX, USA) and a USB-6009 DAQ (National Instruments, Austin, TX, USA).

Each tracking pattern was predetermined before the experiment. This task consisted of a waveform with predictable and unpredictable epochs. The first and third epochs lasted ten s and were unpredictable as they consisted of randomly generated patterns between blocks. The middle segment lasted 10 s and was predictable, comprising the same repeated pattern across trials and blocks. The waveform was generated using a general sine-cosine series [35]:

$$f\left(x\right)=b_{0}+a_{i}\sin\left(x\right)+b_{1}\cos\left(x\right)+a_{2}\sin\left(2x\right)+b_{2}\cos\left(2x\right)+\cdots +a_{6}\sin\left(6x\right)+b_{6}$$

The middle segment was comprised of the same repeated pattern on every trial. The coefficients (b_i_ and a_i_) of the middle-repeated epochs were the same for each trial, consisting of b_0_ = 2.0, a_1_ = −4.0, b_1_ = 3.0, a_2_ = −4.9, b_2_ = −3.6, a_3_ = 3.9, b_3_ = 4.5, a_4_ = 0.0, b_4_ = 1.0, a_5_ = −3.8, b_5_ = −0.5, a_6_ = 1.0, and b_6_ = 2.5 [16]. The outer random epoch coefficients were randomly selected for each block from a set of values between −5 and 5. The waveform of each block was reset after each block. To guarantee that learning was not attributed to the difficulty of the segment, the number of extrema was kept similar across all outer random segments and trials [36]. Lastly, the raw position data was smoothed using a 100-millisecond moving average [6].

Continuous tracking task performance was quantified using the root mean square error (RMSE). RMSE measured overall tracking error as the average difference between the target pattern and the participant’s response across time. RMSE was defined as follows:

$$RMSE=\sqrt{\left(\frac{\sum {\left(y_{i}-{\hat{y}}_{i}\right)}^{2}}{n}\right)}$$

where y_i_ is the cursor position, ŷ_i_ is the target position, and n is the number of samples. The RMSE was calculated separately for repeating sequences and random epochs for each task block. The outer random epochs’ RMSE was averaged to compare with the repeated epoch [37,38].

### 2.4. Delayed Retention Test

The delayed retention test involved performing one block of 12 trials of the continuous tracking task. The delayed retention test was used to determine the extent to which the performance gains were associated with offline consolidation. The retention test took place 24–48 h after the original practice session.

### 2.5. Recognition Test

The recognition test involved the presentation of ten waveform segments. Each segment was 10 s long, the same length as one of the three segments that made up a single trial of practice. Participants watched the waveform segment but did not track it. After the segment was complete, participants were asked if the segment represented something they had practiced. Participants were required to respond “yes” or “no” using a standard computer mouse to click on the corresponding user prompt. Three of the ten segments were the repeated sequence that occurred on every trial of practice. The remaining seven segments were novel foils. Participants were considered to have gained explicit knowledge of the sequence if they recognized two of the three repeated patterns and failed to recognize more than four of the seven foil patterns [6].

The explicit group performed the recognition test immediately after receiving explicit instruction about the repeated sequence in the first session to confirm their explicit knowledge of the sequence. The implicit and explicit groups performed the recognition test immediately following the delayed retention test during the second session. Both groups performed the second session recognition test to assess the extent to which the implicit group autogenously acquired explicit knowledge about the repeating pattern.

### 2.6. Controllable Pulse Parameter Transcranial Magnetic Stimulation (cTMS)

MEPs elicited by cTMS were recorded using LabChart 8 software, a Quad BioAmp and a PowerLab 4/35 acquisition system (AD Instruments, Colorado Springs, CO, USA). Surface electromyography recording was triggered using a 5 V TTL pulse with an epoch of −0.3–0.5 s. Data was amplified (×1000), digitized (×40,000 Hz), and filtered (bandpass filtered 5–1000 Hz, notch filter 60 Hz). The MEP was defined as the peak-to-peak amplitude of the maximal electromyography response between 20 and 50 ms post-TMS stimulation.

TMS was delivered using an Elevate cTMS stimulator (Rogue Research, Montreal, QC, Canada). The current direction was controlled using two different 70 mm medium inductance (20 µH) figure-8 coils. The physical geometry of the coils was identical; however, the wiring of one coil was reversed by the manufacturer so that the direction of the initial positive phase of the electric field induced AP current in the underlying neural tissue. Stimulus duration (e.g., 30 or 120 µs) refers to the duration of the dominant initial positive phase of the TMS pulse and was controlled through the stimulator’s onboard control software. The M-ratio was set to 0.2 [39]. The M-ratio refers to the ratio of the capacitor voltages responsible for the positive and negative field phases of the TMS stimulus [33]. An M-ratio of one represents a balanced biphasic stimulus where the strength of the positive and negative electronic fields is equivalent. Decreasing the M-ratio reduces the magnitude of the biphasic stimulus’s negative (second phase) to introduce an imbalanced biphasic stimulus where the initial positive field phase is responsible for the evoked response [39]. The imbalanced pulse approximates the traditional monophasic while providing the opportunity to reduce the duration of the positive phase. An M-ratio of 0.2 was chosen for the current study, as past work has established that this ratio best approximates a monophasic stimulus [39].

The FDI motor cortical hotspot was defined as the scalp position that elicited the largest and the most consistent MEP to PA_120_ stimulation while the coil was held ~45° to the midline. The coil’s scalp location and trajectory were recorded using the BrainSight™ stereotactic system (Rogue Research, Montreal, QC, Canada). The same hotspot was used for AP_30_ stimulation [34,40]. The motor threshold was defined as the stimulus intensity required to elicit the target MEP amplitude while the participant maintained ~10% of maximal contraction of the FDI. Motor threshold was determined using the maximum likelihood parameter estimated (ML-PEST) adaptive threshold-hunting method [41].

The ML-PEST method uses a binary, yes or no response to model an S-shaped probability function for evoking an MEP above the criterion amplitude. For the AP_30_ stimulus with an M-ratio of 0.2, the maximal stimulator output was 100%. Therefore, the value indicated by the ML-PEST program was entered as the percent of maximal stimulator output at a 1:1 ratio.

All participants maintained a 10% contraction for all thresholding procedures using the force sensor and visual feedback. The motor threshold was first defined for AP_30_ current using the ML-PEST method software [41]. We first attempted to establish the motor threshold using a 1 mV MEP criterion. If a 1 mV MEP could not be achieved for AP_30_ before exceeding maximal stimulator output, the same thresholding procedure was repeated with a 0.5 mV MEP criterion. If a MEP 0.5 mV could not be obtained before exceeding maximal stimulator output, the participant was classified as AP_30_-negative. For those classified as AP_30_-negative, the same thresholding procedure was conducted using PA_120_ current and a 1 mV MEP as the target threshold criterion. For all participants classified as AP_30_-negative, an MEP of 1 mV using PA_120_ current was achieved.

One consideration when using the ML-PEST method with cTMS is that 100% of maximal stimulator amplitude may be unavailable for certain combinations of current direction, positive phase duration, and M-ratio. The maximum stimulus intensity for the PA_120_ pulse with a 0.2 M ratio is 50% of the maximum stimulator output. Therefore, when thresholding PA_120_, ML-PEST intensities were scaled to cTMS stimulus intensity by dividing the ML-PEST value by 2. Thus, 100% for the ML-PEST program equated to 50% of the stimulator output on the cTMS unit.

### 2.7. Data Analysis

Statistical analyses were conducted using the R software (version 4.2.3) and the “rstatix” [42], “tidyverse” [43], and “ggpubr” [44] packages. Q-Q plots and the Shapiro–Wilk test were used to evaluate the normality of the distributions. The assumption of normality and homogeneity of variance were assessed using the Shapiro–Wilks and Levene’s tests, respectively.

Overall performance improvement was quantified as the percent change in RMSE scores between the first block of practice and the delayed retention test. Online learning was quantified as the percent change in RMSE from the first to the last practice block during the first session. Offline consolidation was quantified as the percent change in RMSE from the last block of practice from the first session to the delayed retention test.

First, general improvements in motor ability were assessed by comparing the change in RMSE for the random sequences only. The separate Threshold Response (AP_30_-positive, AP_30_-negative) ×Instruction (Explicit, Implicit) between-subjects analysis of variance (ANOVA) was conducted for overall performance, online learning, and offline consolidation. Where significant interactions were identified, follow-up comparisons were conducted using pairwise contrasts of estimated marginal means with the Bonferroni corrections.

Second, sequence-specific learning was assessed using separate Threshold Response (AP_30_-positive, AP_30_-negative) × Instruction (Explicit, Implicit) × Waveform (Random, Repeated) mixed design ANOVAs for overall performance improvement, online learning, and offline learning. Sequence-specific learning is reflected in the difference in RMSE changes for repeated sequences above and beyond the changes seen for random sequences. In all cases, Threshold Response and Instruction were considered between-subject factors. Waveform was entered as a within-subjects factor.

Significant three-way interactions were decomposed for all analyses using separate Instruction (Explicit, Implicit) × Waveform (Random, Repeated) mixed-design ANOVAs for each Threshold Response group.

## 3. Results

### 3.1. General Improvements in Motor Ability

**Figure 3:**
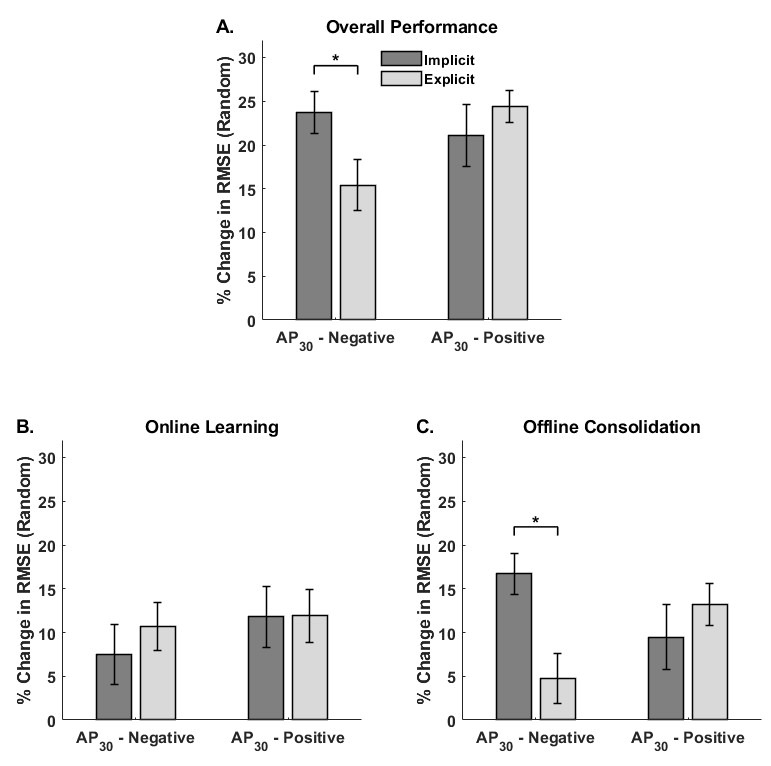
Percent change in root mean square error (RMSE) on the random waveform epochs shown for each Threshold Response and Instruction group across (**A**) overall performance (baseline to delayed retention), (**B**) online learning (within the first training session), and (**C**) offline consolidation (from the end of training to delayed retention). The change in random sequence tracking indexes general motor improvement independent of sequence learning. In all cases, higher values indicated greater improvements in performance. Error bars represent the standard error, and asterisks (*) indicate statistical significance (*p* < 0.05).

Figure 3 illustrates the mean percent change in random sequence tracking performance for overall performance, online learning, and offline consolidation for each Threshold Response and Instruction group.

#### 3.1.1. Overall Performance

The Threshold Response × Instruction ANOVA revealed a significant interaction [F_1,68_ = 4.49, *p* = 0.038, η_p_^2^ = 0.062] (Figure 3A). Post-hoc comparisons indicated that, among the AP_30_-negative participants, the implicit group demonstrated greater improvements than the explicit group in general motor ability from the first block of practice to the delayed retention test (*p* = 0.0046). In contrast, sensorimotor efficiency improved similarly for both the implicit and explicit AP_30_-positive groups (*p* = 0.39).

#### 3.1.2. Online Learning

The Threshold Response × Instruction between-groups ANOVA comparing random sequence tracking performance from the start to end of practice failed to reveal any significant effects [Interaction: F_1,68_ = 0.25, *p* = 0.62, η_p_^2^ = 0.004; Main Effect_Threshold_: F_1,68_ = 0.77, *p* = 0.38, η_p_^2^ = 0.01, Main Effect_Instruction_: F_1,68_ = 0.27, *p* = 0.61, η_p_^2^ = 0.004] (Figure 3B).

#### 3.1.3. Offline Consolidation

The Threshold Response × Instruction ANOVA revealed a significant interaction [F_1,68_ = 7.43, *p* = 0.008, η_p_^2^ = 0.10] (Figure 3C). The main effects of Threshold Response [F_1,68_ = 0.05, *p* = 0.83, η_p_^2^ < 0.001] and Instruction [F_1,68_ = 2.03, *p* = 0.16, η_p_^2^ = 0.03] were not significant. Post hoc comparisons indicated offline consolidation of general sensorimotor improvement was greater for the AP_30_-negative implicit group compared to the AP_30_-negative explicit group (*p* = 0.0046). In contrast, the implicit and explicit AP_30_-positive groups demonstrated similar offline consolidation of general improvements in motor control (*p* = 0.36).

### 3.2. Sequence Specific Learning

#### 3.2.1. Overall Performance

**Figure 4:**
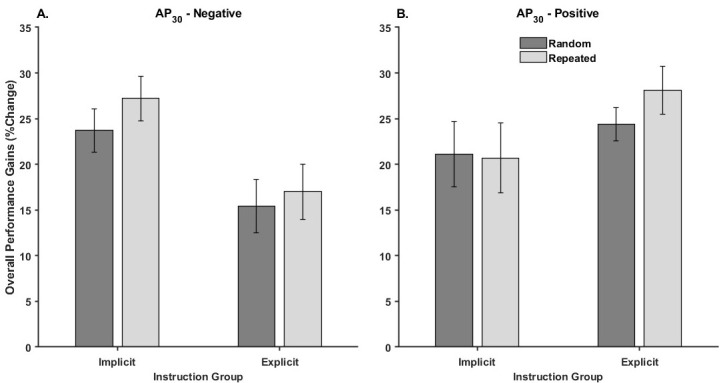
Overall performance change for each instruction group and waveform type for the (**A**) AP_30_-negative and (**B**) AP_30_-positive group. In all cases, higher values indicated greater improvements in performance from baseline testing to the delayed retention test. Error bars represent the standard error.

Figure 4 illustrates the mean percent change in overall performance for each Threshold Response group by Instruction and Waveform.

The Threshold Response × Instruction × Waveform mixed design ANOVA revealed a significant three-way interaction [F_1,68_ = 4.24, *p* = 0.04, η_p_^2^ = 0.06]. There was also a significant Threshold × Instruction interaction and a significant main effect of waveform (Table 2). None of the other effects were significant (Table 2).

Decomposition revealed a strong trend towards a significant Instruction x Waveform interaction in the AP_30_-positive group [F_1,34_ = 3.84, *p* = 0.058, η_p_^2^ = 0.10]. The interaction trend in the AP_30_-positive group was driven by a significantly greater improvement for the repeated sequence compared to the random sequence for the explicit group (*p* = 0.02) but no difference for the implicit group (*p* = 0.78) (Figure 4B). In contrast, the Instruction x Waveform interaction effect size for the AP_30_-negative group was weak and failed to reach significance [F_1,34_ = 0.89, *p* = 0.35, η_p_^2^ = 0.03]. Instead, the main effects of Waveform [F_1,34_ = 6.57, *p* = 0.015, η_p_^2^ = 0.16] and Instruction [F_1,34_ = 6.30, *p* = 0.017, η_p_^2^ = 0.16] were both significant. The main effect of Waveform was driven by greater improvement in repeated sequence tracking (22.1 ± 2.1%) compared to random sequence tracking (19.5 ± 2.0%), regardless of Instruction. The main effect of Instruction was driven by greater improvement in tracking performance for the AP_30_-negative implicit group (25.4 ± 1.7%) compared to the explicit group (16.2 ± 2.1%), regardless of waveform.

#### 3.2.2. Online Learning

**Figure 5:**
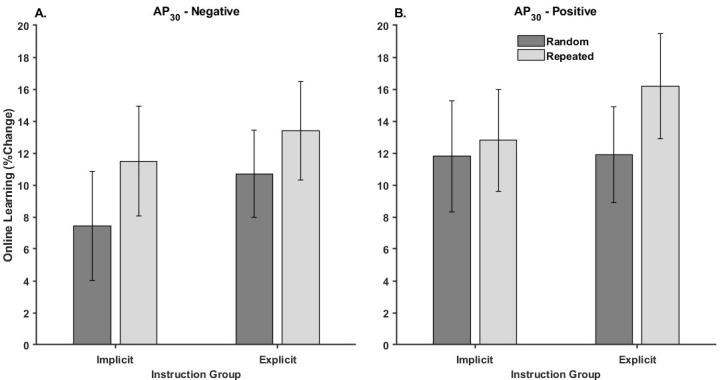
Performance changes due to online learning for each instruction group and waveform for the (**A**) AP_30_-negative and (**B**) AP_30_-positive group. In all cases, higher values indicated greater improvements in performance from baseline testing to the end of the training on the first session.

Figure 5 illustrates the mean percent change in online learning performance for each Threshold Response group by Instruction and Waveform.

The Threshold Response × Instruction × Waveform mixed design ANOVA yielded a significant main effect of Waveform [F_1,68_ = 13.47, *p* < 0.001, η_p_^2^ = 0.17]. None of the other interactions or main effects were significant (Table 3). The main effect of Waveform was driven by a greater increase in tracking performance for the Repeated (13.5 ± 1.6%) compared to the Random (10.5 ± 1.6%) waveform epochs from initial practice to practice block eight within the first session.

### 3.2.3. Offline Consolidation

**Figure 6:**
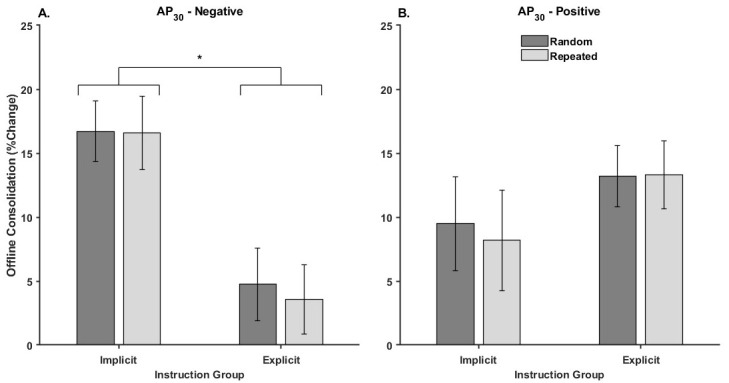
Performance changes due to offline consolidation for each instruction group and waveform for the (**A**) AP_30_-negative and (**B**) AP_30_-positive group. In all cases, higher values indicated greater improvements in performance from the last block of practice in session one to the delayed retention test. Error bars represent the standard error, and the asterisks (*) indicate statistical significance (*p* < 0.05).

Figure 6 illustrates the mean percent change in offline consolidation for each Threshold Response group by Instruction and Waveform.

The Threshold Response × Instruction × Waveform mixed ANOVA on performance gains from the last block of practice to the delayed retention test revealed a significant Threshold Response × Instruction interaction [F_1,62_ = 9.05, *p* = 0.004, η_p_^2^ = 0.12]. None of the other effects were significant (Table 4).

The Threshold Response × Instruction interaction reflects that offline consolidation was similar for random and repeated sequences, indicating gains were in general sensorimotor ability rather than sequence-specific learning. Decomposition of the Threshold Response × Instruction interaction revealed that offline improvements were significantly greater for the Implicit compared to the Explicit group for those who were AP_30_-negative (*p* = 0.000006; Implicit = 17.1 ± 2.1%, Explicit = 3.6 ± 2.0%), regardless of waveform. In contrast, there was no difference in offline improvement between the implicit and explicit groups for those who were AP_30_-positive (*p* = 0.17; Implicit = 8.8 ± 2.7%, Explicit = 13.3 ± 1.8%), regardless of waveform.

## 4. Discussion

The current study investigated the interaction between interneuron excitability and instruction on motor learning following a single training session. The novel finding was that individuals with lower interneuron excitability, represented as higher AP_30_ TMS thresholds (AP_30_-negative), demonstrated less sustained improvement in continuous visuomotor tracking performance when provided explicit instruction than those who practiced under implicit conditions. The effect of explicit instruction in the AP_30_-negative group appears to be tied to reduced offline consolidation of general sensorimotor ability rather than sequence-specific elements of the task. In contrast, for individuals with lower AP_30_ TMS thresholds (AP_30_-positive), improvements in general sensorimotor ability were not sensitive to the explicit or implicit nature of the task. However, the AP_30_-positive explicit group did demonstrate greater sequence-specific learning than the AP_30_-positive implicit group. These results support the hypothesis that those with low and high AP_30_ intraneuronal excitability responded differently to instruction during the early stages of motor learning.

The current study was unique as participants were grouped based on a neurophysiological measure, AP_30_ interneuron circuit excitability. The differential sensitivity to explicit instruction between the AP_30_-positive and AP_30_-negative groups may help explain some of the variability within and across behavioral studies investigating the effect of instruction on motor learning in healthy young adults [11,45]. Studies failing to demonstrate differences between explicit and implicit instruction may reflect a heterogeneous mix of AP_30_ excitability. Those who are AP_30_-positive, indicative of greater excitability in the motor cortex substrates recruited by the AP_30_ TMS stimulus, may be better positioned to balance explicit knowledge with implicit processes during offline consolidation [9]. In contrast, those who are AP_30_-negative demonstrate interference between explicit and implicit processes during offline consolidation. The observed effect of offline consolidation primarily involved sensorimotor efficiency, with no reliable modulation of sequence-specific learning. This suggests that explicit knowledge more robustly disrupted the retention of general motor control processes rather than sequence-specific learning [6].

From a neurophysiological standpoint, the dissociation between the AP_30_-positive and AP_30_-negative groups is consistent with functional connectivity [30], computational modelling [46], and afferent inhibition studies [47], suggesting the pathways involving the interneurons recruited by AP TMS current originate in premotor regions. Volz et al. [30] associated the response to the AP TMS stimulus with variability in premotor-M1 functional connectivity. The relationship between premotor-M1 structure and functional connectivity in motor skill learning is well-established in many populations that have achieved optimal performance and automaticity. For example, professional musicians have greater cortical thickness in premotor, motor, and somatosensory areas [48,49] and experience-dependent increases in myelination in white matter tracts [50]. Similar experience-dependent structural and functional differences are seen in other forms of repetitive, intentional sensory-motor training, such as sports [51,52]. Thus, the negative impact of explicit instruction specific to the AP_30_-negative group is consistent with weaker functional connectivity in procedural sensorimotor networks, leaving them susceptible to explicit instruction during early learning. In contrast, enhanced connectivity allowed those in the AP_30_-positive group to maximize procedural learning without as much interference from explicit knowledge and strategies. The ability to exploit the explicit instruction without sacrificing procedural elements of skill learning is further supported by the absence of sequence-specific learning in the implicit AP_30_-positive group from the start of practice to the delayed retention test. While this group demonstrated similar improvements in general motor ability across this period, they did not demonstrate the ability to extract sequence-specific knowledge in the absence of being made aware that there was a sequence embedded in the tracking task. Yet the AP_30_-positive explicit group demonstrated similar improvements in general motor ability and concurrently developed sequence-specific knowledge. Interestingly, offline consolidation of general motor ability in the AP_30_-negative implicit group was qualitatively greater than in all other groups, further supporting the notion that explicit instruction interfered with the consolidation of implicit procedural processes in the AP_30_-negative explicit group.

The relative importance of the excitability of the substrates recruited by AP_30_ TMS stimuli to sensorimotor learning may be linked to the modulation of somatosensory afferent projections to premotor-M1 pathways via AP_30_-sensitive inputs [47,53]. Central integration of somatosensory afference is critical to learning [54,55,56] and may be emphasized during non-ballistic, graded motor skills like the continuous tracking task employed in the current study. Modulation of the frontal P20-N30 somatosensory evoked potential is shown to parallel modulation of AP-sensitive circuits under varying attentional load [47,53]. The frontal P20-N30 generators are localized to premotor and supplementary motor areas of the precentral gyrus [57]. Modulation of these projections onto AP-sensitive corticospinal inputs would be consistent with computational models suggesting AP-induced current primarily activates axon terminals in the gyral crown near the premotor regions [46] and the association between AP current response and the functional connectivity between the primary motor cortex and premotor areas [30].

Another interesting finding from the current study is the dissociation of general sensorimotor ability and sequence learning across AP_30_ excitability depending on instruction. For general sensorimotor ability, the differences in overall performance from the start of practice to the delayed retention test were driven by a strong reduction in the ability of the AP_30_-negative explicit group to consolidate general sensorimotor abilities. In contrast, for sequence-specific learning, the differences in overall performance from the start of practice to the delayed retention test were associated with weaker sequence-specific learning in the AP_30_-positive implicit group. Qualitatively, the driving force behind the sequence-specific learning effects appears to be related to online learning rather than offline consolidation. However, the sequence-specific learning effect during online learning is relatively weaker than the offline consolidation effect on general sensorimotor ability. The weaker effect of AP_30_ sensorimotor loop excitability as a modulatory influence on sequence-specific learning during online learning is consistent with past experience-dependent dissociations between PA and AP CBI during learning [58]. One possibility is that online learning depends on autogenously forming explicit stimulus-response associations. Somatosensory projections to the motor substrates recruited by the PA stimulus are modulated depending on the relevance of a given muscle to the impending movement [59,60] and its relative position within a sequence [61]. The sensitivity of the PA sensorimotor loop has led to speculation that this loop plays a role in action selection through surround inhibition [62] or movement-related gating [63]. The association of PA SAI with the early parietal N20-P25 somatosensory evoked potential [64] is consistent with a prefrontal-thalamic gating mechanism to prime action selection [65] that is sensitive to attentional focus [61]. PA thresholds are universally lower than AP thresholds, and the I-waves generated by the PA stimulus are also less variable [24]. Therefore, the similarity between groups defined by the AP threshold likely reflects greater homogeneity of PA excitability as both the explicit and implicit groups developed declarative knowledge of the skill exogenously and autogenously during practice. Unfortunately, a limitation of the current study is that we cannot investigate the relationship between PA_120_ thresholds and online sequence-specific learning. While we did obtain the PA_120_ 1 mv threshold for those who were AP_30_-negative, we did not obtain the PA_120_ 1 mv threshold for those who were AP_30_-positive.

The current study has additional potential limitations that need to be acknowledged. First, it is essential to note that we examined early learning during a single session. Thus, our results reflect the early stages of skill acquisition. Early stages of learning are characterized by the recruitment of broad cortico-striatal, cortico-cerebellar, and cortico-cortical substrates, including frontal associative areas like the dorsolateral prefrontal cortex [18]. As learning progresses, there is a shift to cortico-striatal and cortico-cerebellar systems depending on the nature of the skill (acquisition vs. maintenance) [18]. Further, the relative importance of the frontal cortico-cortical projections declines [18]. Therefore, the interaction between the AP_30_ threshold and explicit knowledge may not represent more extensive training that lasts multiple days.

A second limitation is that, while we establish cause and effect, we cannot quantify the relationship between AP_30_ excitability and motor learning because we cannot establish an AP_30_ threshold before reaching maximum stimulator output. It is not uncommon that an AP threshold cannot be elicited in all participants before exceeding maximal stimulator output, especially for short-duration AP currents, even with a slight contraction of the target muscle [34,66]. Thus, we could only obtain an AP_30_ threshold for those with relatively higher AP_30_ excitability. Whether the effect of AP_30_ excitability is graded or all-or-none could not be established, as any attempted correlation would be missing data for relatively lower levels of AP_30_ excitability.

## 5. Conclusions

The current study illustrates that AP_30_ interneuron circuit excitability reflects variations in the sensorimotor network that dictate how explicit instruction modulates procedural sensorimotor learning during early practice. Extensive explicit knowledge negatively impacts individuals with less excitable AP_30_ interneuron circuits. In contrast, individuals with more excitable AP_30_ interneuron circuits are better positioned to use explicit knowledge to their advantage. The different responses to explicit instruction likely reflect the resiliency of procedural premotor-primary motor functional networks, consistent with the idea that these networks are critical to experience-dependent plasticity. These findings emphasize the importance of AP_30_ interneuron excitability in understanding sensorimotor learning and control.

## Figures and Tables

**Table 1:** Participant characteristics for the AP_30_-positive and AP_30_-negative groups based on instruction. Sample size (N) and biological sex are counts. Mean and standard deviation (in parentheses) are reported for age and TMS threshold.

Group ^1^	N	Sex	Age	AP_30_ TMS Threshold	PA_120_ TMS Threshold
AP_30_-positive					
Implicit	18	6 M, 12 F	22.3 ± 4.9	82.6 ± 11.0	-
Explicit	18	8 M, 10 F	24.8 ± 4.9	80.3 ± 10.3	-
AP_30_-negative					
Implicit	18	8 M, 10 F	22.6 ± 4.4	-	31.0 ± 10.0
Explicit	18	10 M, 8 F	23.0 ± 3.7	-	33.2 ± 5.1

^1^ AP_30_-positive indicated the ability to establish an AP_30_ TMS threshold before reaching maximal stimulator output. AP_30_-negative indicates the group in which an AP_30_ threshold could not be established before reaching maximal stimulator output.

**Table 2:** ANOVA summary table for the effects of threshold response, instruction, and waveform on overall performance change scores. This table shows the results of a three-way ANOVA, examining the effects of threshold response, instruction, and waveform as their interactions on overall performance change scores. Significant main effects and interactions at *p* < 0.05 are indicated with an asterisk (*).

Effect	df_n_	df_d_	F	*p*	*p* < 0.05	η_p_^2^
Threshold response	1	68	0.97	0.33		0.01
Instruction	1	68	0.48	0.49		0.01
Waveform	1	68	8.40	0.01	*	0.11
Threshold response × Instruction	1	68	6.84	0.01	*	0.09
Threshold response × Waveform	1	68	0.47	0.49		0.01
Instruction × Waveform	1	68	0.55	0.46		0.01
Threshold response × Instruction × Waveform	1	68	4.24	0.04	*	0.06

**Table 3:** ANOVA summary table for the effects of Threshold response, Instruction, and Waveform on online performance change scores. This table shows the results of a three-way ANOVA, examining the effects of Threshold response, Instruction, and Waveform as their interactions on overall performance change scores. Significant main effects at *p* < 0.05 are indicated with an asterisk (*).

Effect	df_n_	df_d_	F	*p*	*p* < 0.05	η_p_^2^
Threshold response	1	68	0.60	0.44		0.01
Instruction	1	68	0.48	0.49		0.01
Waveform	1	68	13.47	<0.001	*	0.17
Threshold response × Instruction	1	68	0.02	0.89		<0.001
Threshold response × Waveform	1	68	0.21	0.65		0.003
Instruction × Waveform	1	68	0.39	0.54		<0.001
Threshold response × Instruction × Waveform	1	68	1.99	0.16		0.043

**Table 4:** ANOVA summary table for the effects of Threshold Response, instruction, and waveform on overall performance change scores. This table shows the results of a three-way ANOVA, examining the effects of Threshold Response, instruction, and waveform as their interactions on overall performance change scores. Significant interactions at *p* < 0.05 are indicated with an asterisk (*).

Effect	df_n_	df_d_	F	*p*	*p* < 0.05	η_p_^2^
Threshold response	1	68	0.05	0.82		0.001
Instruction	1	68	2.06	0.16		0.029
Waveform	1	68	0.39	0.54		0.006
Threshold response × Instruction	1	68	9.05	0.004	*	0.12
Threshold response × Waveform	1	68	<0.001	0.10		<0.001
Instruction × Waveform	1	68	0.004	0.95		<0.001
Threshold response × Instruction × Waveform	1	68	0.41	0.53		0.006

## Data Availability

The raw data supporting the conclusions of this article will be made available by the authors on request.
